# Supplementary material for: Reaction Pathways for the Enzymatic Degradation of Poly(Ethylene Terephthalate): What Characterizes an Efficient PET‐Hydrolase?
Source: Chembiochem. 2022 Dec 12;24(3):e202200516. doi: 10.1002/cbic.202200516 (PMC10108200; doi:10.1002/cbic.202200516)
Supplement: Supplementary file 1 — Supporting Information [file CBIC-24-0-s001.pdf]

ChemBioChem

Supporting Information

Reaction Pathways for the Enzymatic Degradation of Poly(Ethylene Terephthalate): What Characterizes an Efficient PET-Hydrolase?

Sune Schubert, Kay Schaller, Jenny Arnling Bååth, Cameron Hunt, Kim Borch, Kenneth Jensen, Jesper Brask, and Peter Westh*

1. ***Figure S1.*** *Progression curves for the initial stage of the reaction*
2. **Table S1.** *Steady state kinetics on soluble reaction fragments*
3. ***Figure S2.*** *Rate of autohydrolysis of ETE and ET*
4. ***Figure S3.*** *Results for the stochastic models fitted to experimental results for IsP and TfC*
5. ***Table S2.*** *Parameters for the stochastic model derived by differential evolution.*
6. ***Figure S4***. *Exemplary Sankey plot for HiC*
7. **Figure S5** *Investigating the Lag-phase for LCC*
8. **Figure S6** *Characterization of TETET*
9. **Figure S7** *Characterization of TETE*

Initial stage of the reaction

Fig. 3 in the main section shows the total concentration of dissolved C_{Teq} (see Eq. 2) as a function of proton release. This representation of the data disguises the enzyme efficiency by not reflecting any given time dependency. The figure below shows the progress curves for both C_{Teq} and the release of protons as function of time.

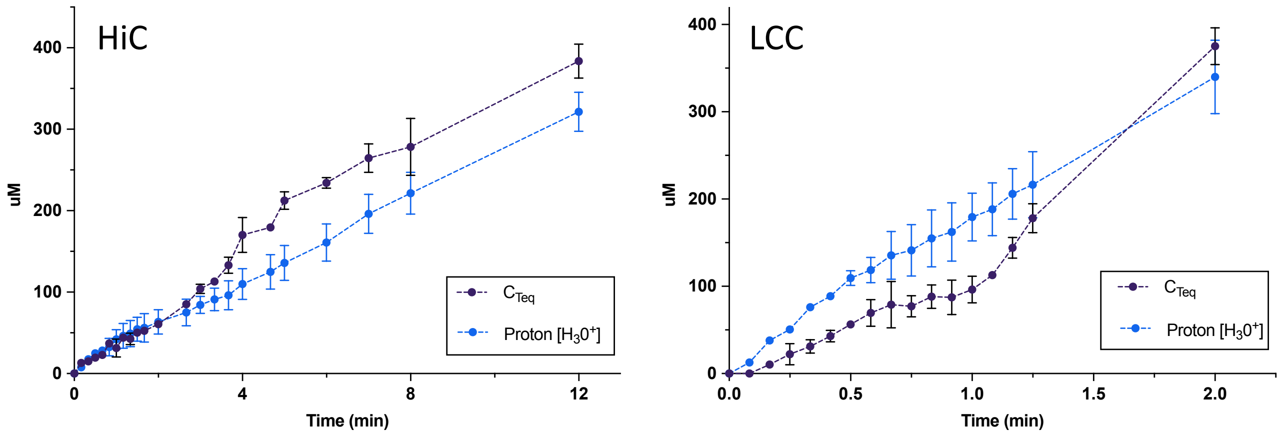

Figure S1. *Progression curves for the initial stage of the reaction for both terephthalic acid equivalents, C_{Teq} (see Eq. 2) and release of protons plotted as a function of time. The results are derived from measurements by respectively pH-stat and RP-HPLC. Error bars represent the standard deviation of duplicate measurements.*

Table S1. *Steady state kinetics on soluble reaction fragments. k_{cat} and K_M values were determined for HiC, TfC, IsP and LCC at an experimental temperature of 50 ˚C. Standard deviations of duplicate measurements are shown in brackets.*

	HiC		TfC		IsP		LCC	
**Substrate**	K_M (mM)	k_{cat} (s^{-1})	K_M (mM)	k_{cat} (s^{-1})	K_M (mM)	k_{cat} (s^{-1})	K_M (mM)	k_{cat} (s^{-1})
ET	**ND**[a]	**ND**	**3.8**[b] (± 0.091)	**0.043** (± 8.5E-4)	**ND**	**ND**	**1.2**[b] (± 0.13)	**0.048** (± 0.0033)
ETE	**3.7** [b]* (± 0.24)	**2.0** (± 0.092)	**0.23** (± 0.067)	**1.1** (± 0.12)	**1.4** [b]* (± 0.082)	**4.8** (± 0.15)	**0.96** [b] (± 0.16)	**6.2** (± 0.62)
TETE	**0.077** (± 0.0091)	**2.9** (± 0.098)	**0.17** (± 0.016)	**11** (± 0.35)	**0.14** (± 0.036)	**14** (± 1.3)	**0.12** (± 0.035)	**21** (± 1.7)
TETET	**0.089** (± 0.021)	**24** (± 2.9)	**0.17** (± 0.038)	**62** (± 7.3)	**0.058** (± 0.029)	**23** (± 4.2)	**0.79**[b] (± 0.41)	**300** (± 122)

a) ND = Not detected
b) Not saturated

*Data from Arnling Bååth et al. 2020 (ChembioChem)

Autohydrolysis of MHET of BHET

While developing the stochastic model intended to examine the enzymatic degradation pathway of PET towards soluble monomers, we obtained results suggesting that the contribution from enzymatic activity alone, could not account for the presence of TPA. Hence, an experiment was conducted to investigate the rate of autohydrolysis of ET and ETE under the conditions used to obtain experimental results for the enzymatic degradation course of PET (see Figs. 1-2). The autohydrolysis of ET and ETE was examined for two initial concentrations that were in proximity to the amount of the respective product detected during our experimental runs (see Fig. 1).

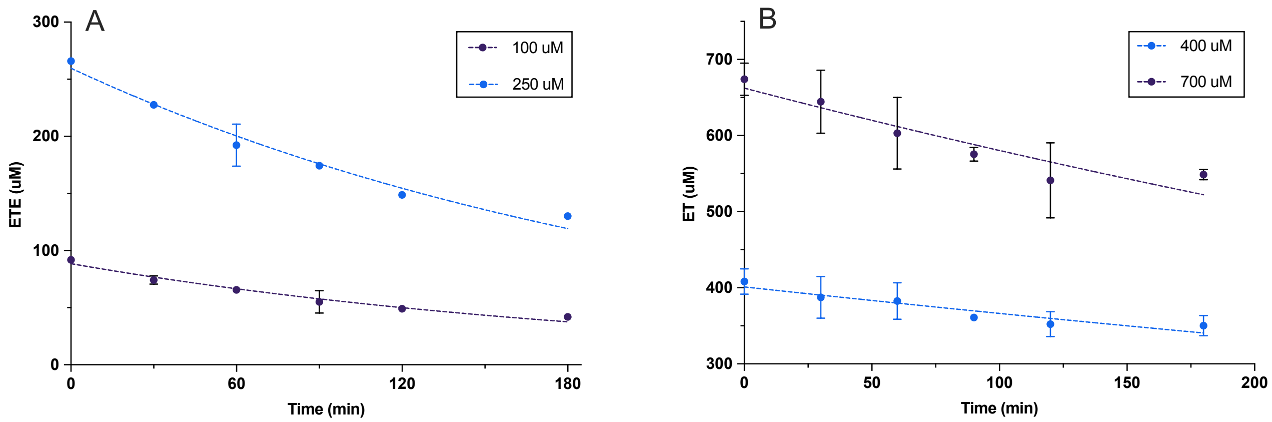

Figure S2. *Rate of autohydrolysis of ETE (A) and ET (B) examined under the same experimental conditions used to obtain the results shown in Figs. 1 and 2. RP-HPLC analysis was used to detect the presence of either ET and ETE and the autohydrolysis of the respective product was tracked over time. Based on our results, the half-life of ETE and ET was estimated to 153.0 ± 10.3 min and 643 ± 168.3 min, respectively.*

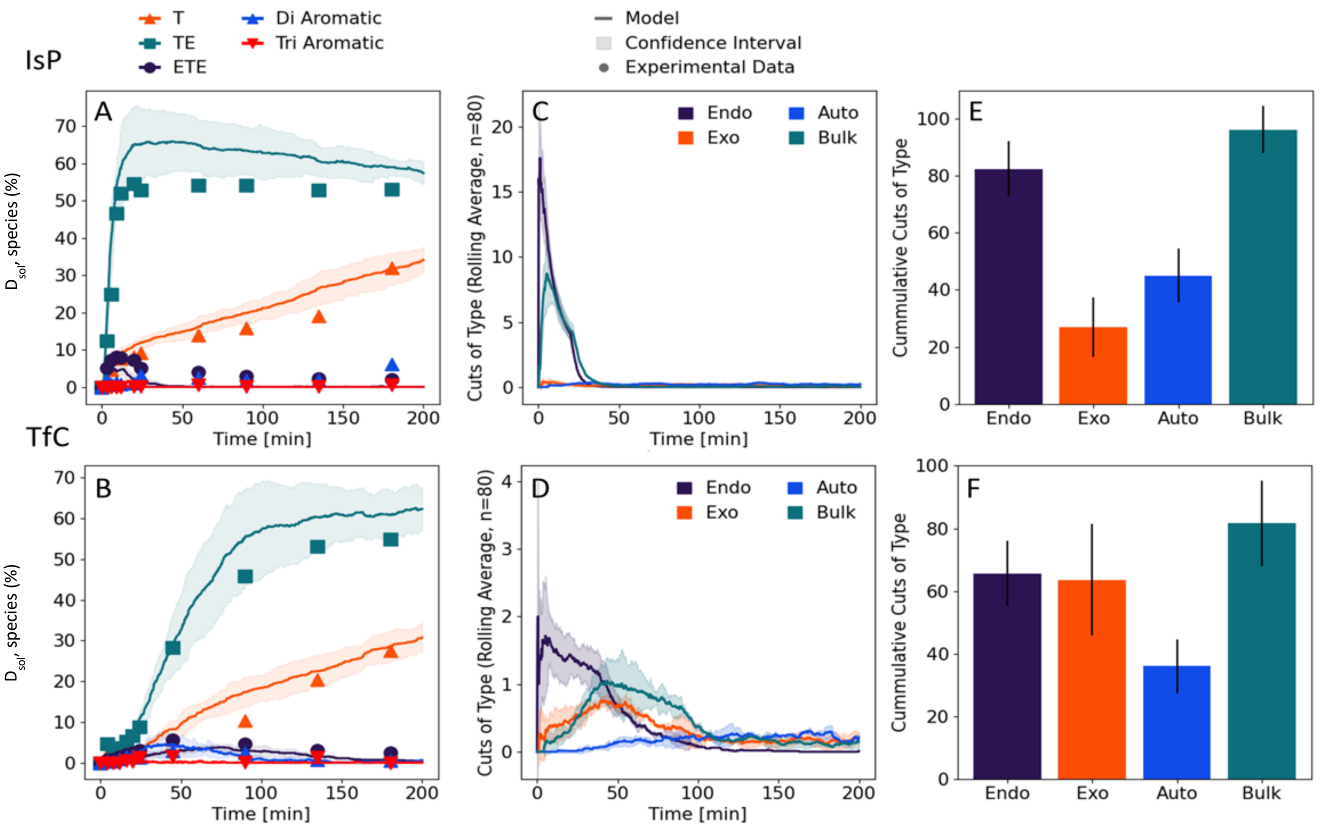

Figure S3. *Results for the stochastic model fitted to experimental results for IsP (top panels) and TfC (lower panels). Average values and standard deviation of 5 repetitions of the stochastic models are shown. A/B) Values corresponding to terephthalic acid equivalents* C_{Teq} *(see Eq. 2) occurring in each species during the reaction. C/D) Mode of hydrolytic event occurring over the time course of the stochastic model. E/F) Accumulated number of cuts over the whole simulation for each of the four reaction types defined in the model (i.e., endolytic, exolytic, bulk (homogenous catalysis) and autohydrolysis (non-enzymatic hydrolysis)***.***

Table S2. *Parameters for the stochastic model derived by differential evolution. The parameters are probabilities per minute to perform a certain type of esterolytic activity. Endo-lytic (p_{endo}) and exo-lytic (p_{exo}) activities on the insoluble substrate as well as enzymatic hydrolysis of solubilized fragments and autohydrolysis of ETE and TE fragments were distinguished (see Fig. 4). The probability of the enzyme to act on soluble fragments (p_{solu}) is weighted with the specificity constant (see Tab. 1), which varies for each enzyme. The probability of autohydrolysis (p_{auto}) was treated as a global parameter and not fitted to each enzyme individually.*

**Enzyme**	p_{endo} [-]	p_{exo} [-]	p_{solu} [-]	p_{auto} [-]
HiC	1.2%	0.7%	0.31%	0.19%
LCC	12.5%	1.1%	0.12%	0.19%
TfC	0.6%	0.9%	0.12%	0.19%
IsP	6.6%	0.7%	0.11%	0.19%

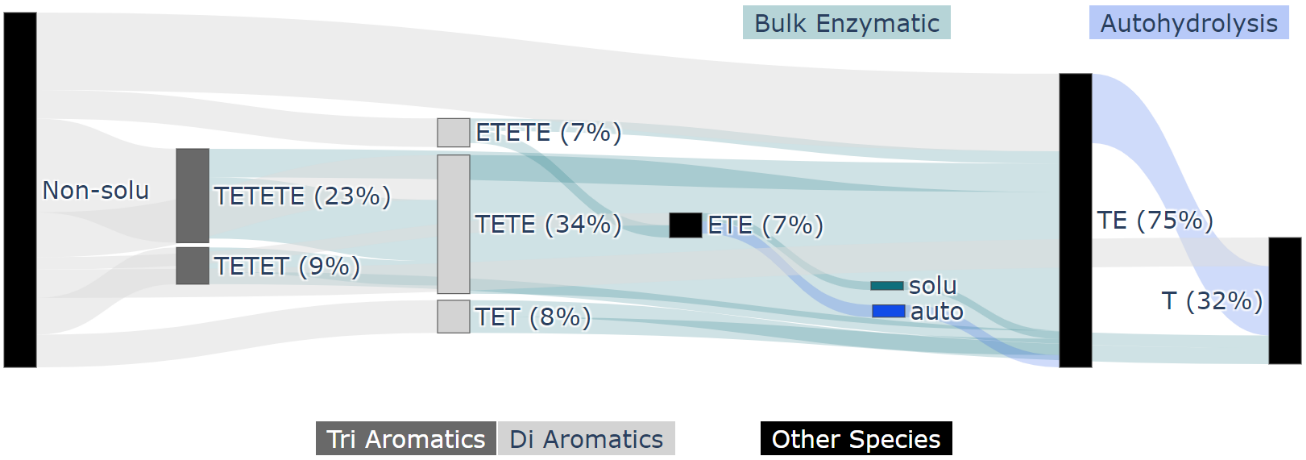

Figure S4. *Exemplary Sankey plot for HiC depicting the degradation pathway from insoluble PET towards monomers over the reaction course elucidated by the stochastic model. Different modes of action and soluble PET fragments are highlighted. The flux of terephthalic acid equivalents (C_{Teq}, see Eq. 2) is scaled to the relative strength of the depicted linkage, and the percentage of the total C_{Teq} flowing through a node is highlighted.*

Investigating the Lag-phase for LCC

Unlike LCC and IsP, the less proficient PET-hydrolases HiC and TfC showed a distinctive lag-phase. An additional experiment was conducted for LCC in which the enzymes dosage was lowered (20 nM and 5 nM) to investigate whether the time-resolution shown in Fig 2 (300 nM LCC) was insufficient to detect the presence of a lag-phases at higher reaction rates.

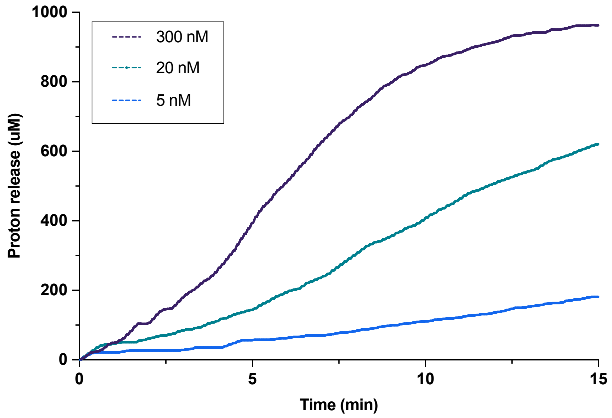

Figure S5*. Progress curves for the release of protons during hydrolysis of nanoPET by 5 nM, 20 nM, and 300 nM LCC.*

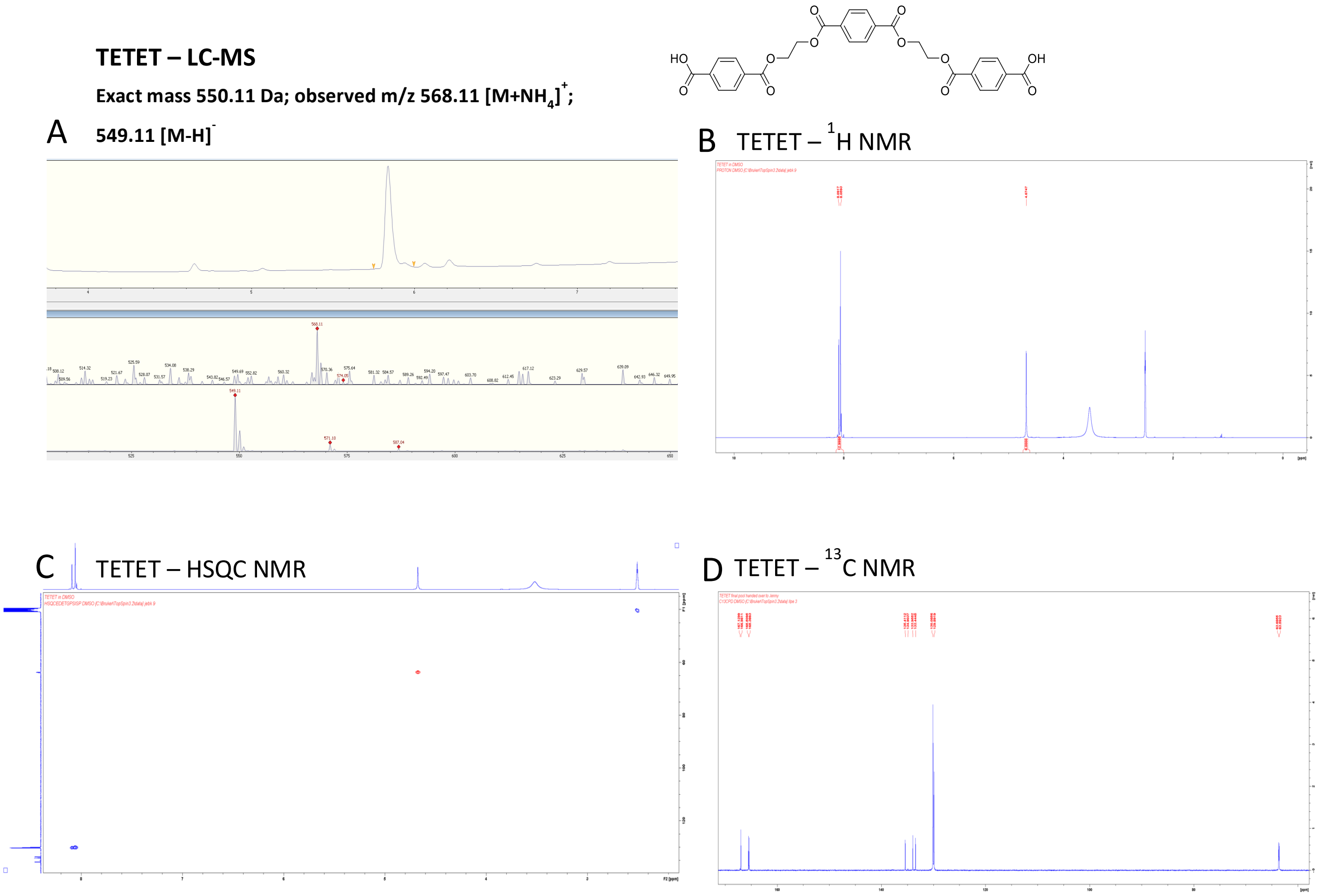

***Figure S6**. Characterization of TETET. A) LC-MS and B)* ^{1}H NMR, C) HSMQ and D) ^{13}C NMR spectra for the in-house synthesized PET fragment; TETET.

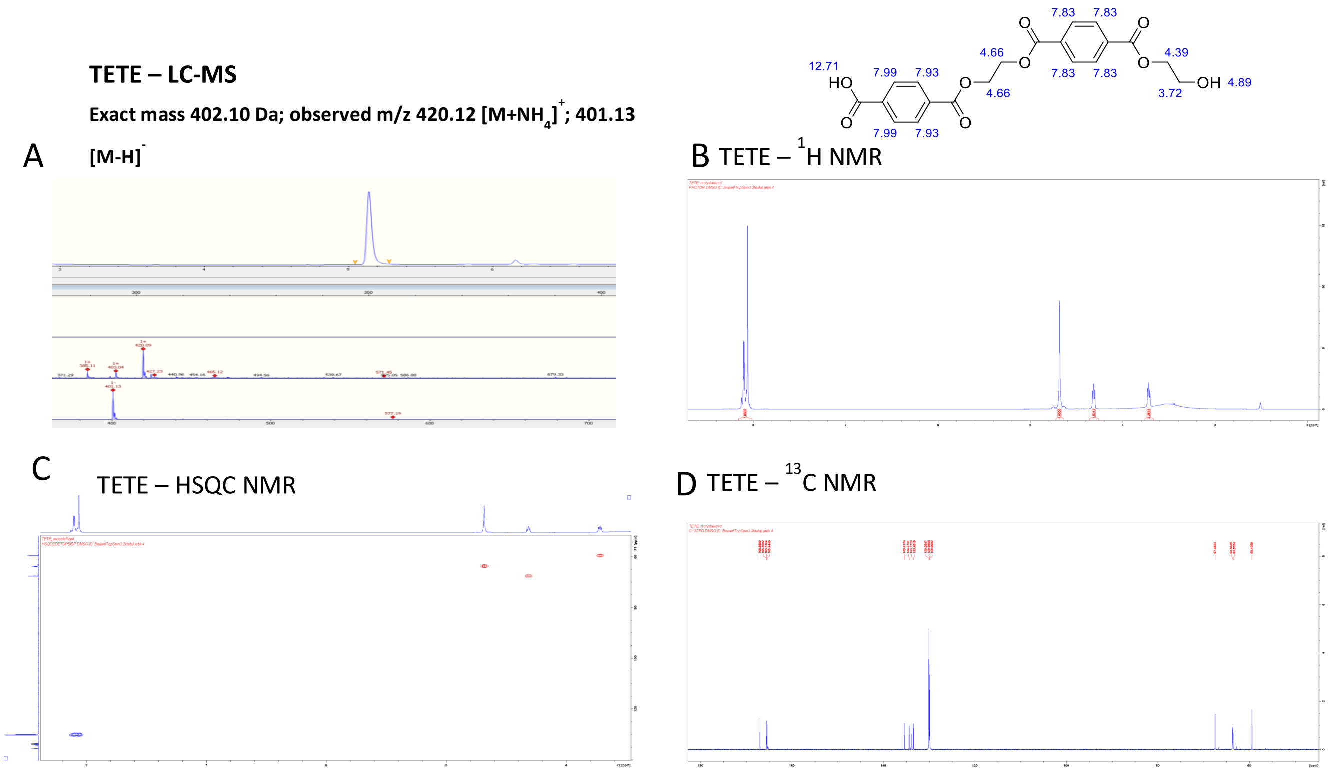

Figure S7*.Characterization of TETE. A) LC-MS and B)* ^{1}H NMR, C) HSMQ and D) ^{13}C NMR spectra for the in-house synthesized PET fragment; TETE.
